# Development of a TGF-β signaling-related genes signature to predict clinical prognosis and immunotherapy responses in clear cell renal cell carcinoma

**DOI:** 10.3389/fonc.2023.1124080

**Published:** 2023-01-27

**Authors:** Xin Wu, Wenjie Xie, Binbin Gong, Bin Fu, Weimin Chen, Libo Zhou, Lianmin Luo

**Affiliations:** Department of Urology, The First Affiliated Hospital of Nanchang University, Nanchang, Jiangxi, China

**Keywords:** TGF-β signaling, clear cell renal cell carcinoma, prognosis signature, immune infiltration, biomarkers

## Abstract

**Background:**

Transforming growth factor (TGF)-β signaling is strongly related to the development and progression of tumor. We aimed to construct a prognostic gene signature based on TGF-β signaling-related genes for predicting clinical prognosis and immunotherapy responses of patients with clear cell renal cell carcinoma (ccRCC).

**Methods:**

The gene expression profiles and corresponding clinical information of ccRCC were collected from the TCGA and the ArrayExpress (E-MTAB-1980) databases. LASSO, univariate and multivariate Cox regression analyses were conducted to construct a prognostic signature in the TCGA cohort. The E-MTAB-1980 cohort were used for validation. Kaplan-Meier (K-M) survival and time-dependent receiver operating characteristic (ROC) were conducted to assess effectiveness and reliability of the signature. The differences in gene enrichments, immune cell infiltration, and expression of immune checkpoints in ccRCC patients showing different risks were investigated.

**Results:**

We constructed a seven gene (PML, CDKN2B, COL1A2, CHRDL1, HPGD, CGN and TGFBR3) signature, which divided the ccRCC patients into high risk group and low risk group. The K-M analysis indicated that patients in the high risk group had a significantly shorter overall survival (OS) time than that in the low risk group in the TCGA (*p* < 0.001) and E-MTAB-1980 (*p* = 0.012). The AUC of the signature reached 0.77 at 1 year, 0.7 at 3 years, and 0.71 at 5 years in the TCGA, respectively, and reached 0.69 at 1 year, 0.72 at 3 years, and 0.75 at 5 years in the E-MTAB-1980, respectively. Further analyses confirmed the risk score as an independent prognostic factor for ccRCC (*p* < 0.001). The results of ssGSEA that immune cell infiltration degree and the scores of immune-related functions were significantly increased in the high risk group. The CIBERSORT analysis indicated that the abundance of immune cell were significantly different between two risk groups. Furthermore, The risk score was positively related to the expression of PD-1, CTLA4 and LAG3.These results indicated that patients in the high risk group benefit more from immunotherapy.

**Conclusion:**

We constructed a novel TGF-β signaling-related genes signature that could serve as an promising independent factor for predicting clinical prognosis and immunotherapy responses in ccRCC patients.

## Introduction

Renal cell carcinoma (RCC) ranked third in aspect of new cases of the genitourinary cancer, and its mortality rate also ranked third among genitourinary cancer. In 2020, there were approximately 431,288 newly diagnosed cases and 179,368 deaths in the world (1). Clear cell renal cell carcinoma (ccRCC) is the most frequently diagnosed histologic type, accounting for approximately 80% of primary RCC (2). At present, the main treatment for localized ccRCC are nephrectomy partially and radically and show favorable efficacy. However, approximately 20-30% of patients are advanced RCC at first visit, with extremely poor overall prognosis (3). Moreover, 20-30% of diagnosed RCC with T1-2 stage would experience tumor metastasis within 1 to 2 years after surgery (4). In recent years, the clinical treatment strategies for advanced ccRCC has evolved greatly, with the emergence of molecule targeted therapy and immune checkpoint therapy (5, 6). In addition, there remains a significant number of the patients with no response or resistance to molecule targeted therapy or immune checkpoint therapy (7, 8). Indeed, it is a huge challenge of clinical work to identify risk stratification in ccRCC patients and optimize individualized therapeutic strategies. Some studies indicated that prognostic models can be used for optimizing risk stratification, providing more accurate clinical treatment and predicting clinical outcome (9, 10). Therefore, identification of reliable prognostic models are especially important to predict clinical outcome and better guide the treatment for ccRCC.

The transforming growth factor (TGF)-β signaling pathway induces a dual role during the development of tumorigenesis. In early stage tumors, TGF-β signaling pathway could induce cell arrest and promote apoptosis, thus serving as a tumor-suppressor. In contrast, in advanced cancer, TGF-β signaling pathway activation could promote tumor progression through inducing cancer cell migration, invasion, epithelial-mesenchymal transition (EMT), and chemical resistance, thus acting as a carcinogenesis factor (11, 12). Several studies have reported that targeting TGF-β pathway could inhibit ccRCC invasion and metastasis *in vitro* and vivo (13, 14). In recent years, with the increasing development of bioinformatics, the use of TGF-β signaling pathway-related genes signature as biomarker and prognostic models in malignant tumor has attracted wide attention. Liao et al. established 8-gene signature as a risk model based on TGF-β signaling pathway-related genes to predict prognosis and immunotherapy of liver hepatocellular carcinoma (15). In addition, Yu et al. developed a 5-gene prognostic model based on TGF-β signaling-related genes to evaluate the clinical outcomes, immunotherapy response and targeted therapy of lung adenocarcinoma (16). However, the TGF-β signaling pathway-related genes prognostic model for ccRCC is still lacking and needs to be further addressed.

In this study, TGF-β signaling-related genes were used to investigate the clinical value of these genes expression profile in ccRCC. A novel risk model based on TGF-β signaling-related genes was constructed using TCGA database and validated in the E-MTAB-1980 database. Then, the risk model effectively divided ccRCC patients into high risk and low risk groups. Overall survival (OS) time was significantly reduced in the high risk group than in the low risk group. Moreover, we investigated the differences between different risk groups among clinicopathological features, immune cell infiltration, and expression of immune checkpoints.

## Material and methods

**Figure 1:**
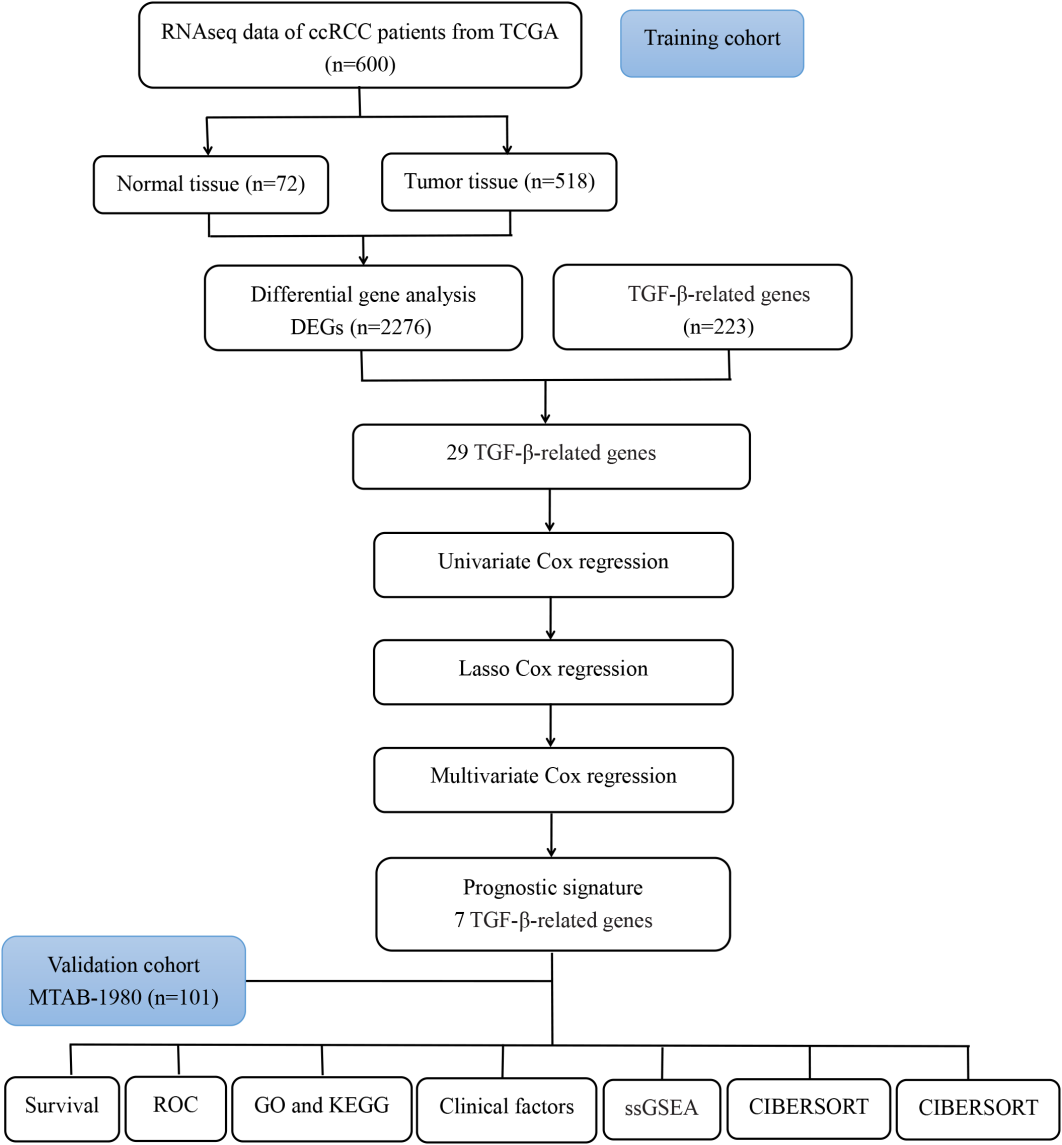
Flow chart of the analysis process in our study.

The flow chart of our study is presented in **Figure 1**.

### Data acquisition

For training cohort, RNA expression data of ccRCC and the corresponding clinical data were collected from TCGA (https://genomecancer.ucsc.edu). For validation cohort, E-MTAB-1980 dataset was collected from ArrayExpress database (https://www.ebi.ac.uk/arrayexpress/). For clinical data, patients who survived less than one month were excluded for subsequent study.

### Identification of TGF-β signaling-related genes

Currently, TGF-β signaling-related genes is lack of comprehensive summary. Thus, TGF-β signaling-related genes were systematically searched from the following databases: AmiGO 2 (http://amigo.geneontology.org/amigo/landing), Ensembl Genome Brower (http://grch37.ensembl.org/index.html) and GSEA (http://www.gsea-msigdb.org/gsea/index.jsp). Finally, a total of 223 TGF-β signaling-related genes were identified in this study **(** **Supplementary Table 1** **).**

### Screening for TGF-β signaling-related differentially expressed genes

The Package “limma” was applied to find TGF-β signaling-related differentially expressed genes (DEGs) between tumor tissues and normal tissues according to the threshold set at |log_2_FC| > 1 and adjusted *P* < 0.05.

### Prognostic gene signature construction and validation

Firstly, we preliminarily determined the TGF-β signaling-related genes affecting OS in TCGA database by univariate Cox analysis. Then, the prognostic genes get from univariate Cox analysis were identified with the Least Absolute Shrinkage and Selection Operator (LASSO) regression in order to avoid overfitting. After that, the candidate genes identified from LASSO analysis were further determined by multivariate Cox regression analysis in order to develop prediction model. The risk model was established based on the following equation: risk score =β_mRNA1_×Expression_mRNA1_+β_mRNA2_×Expression_mRNA2_+β_mRNA3_×Expression_mRNA3_+…+ β_mRNAn_×Expression_mRNAn_.

Next, the risk score of patients was obtained, and patients were assigned to high risk group and low risk group according to the medium value of risk score. K-M method was used to determine the difference of OS between high risk and low risk groups. Finally, ROC curve analysis was used to identify the effectiveness of the risk model.

### Development and evaluation of a predictive nomogram

Based on TGF-β risk score and clinicopathologic features, the univariate and multivariate Cox regression analyses were performed to identify the independent prognostic factors. Then, we integrated the independent prognostic factors to develop a comprehensive nomogram. Furthermore, the effectiveness performance of the nomogram was assessed by calibration curves with “rms” R package.

### Comprehensive analysis of the prognostic model

The relationship between the risk score and clinicopathological features were determined to further evaluate the statistical performance of the prognostic model during the ccRCC development.

### GO and KEGG enrichment analysis

Based on the threshold set at |log_2_FC| > 0.8 and adjusted *P* < 0.05, the Package “limma” was used to identify the risk score-related DEGs. Gene Ontology (GO) analysis and Kyoto Encyclopedia of Genes and Genomes (KEGG) pathway analysis were applied for investigating the biological function of DEGs.

### Evaluation of tumor immune microenvironment

To investigate the difference of infiltrating score between high risk and low risk groups, the single-sample gene set enrichment analysis (ssGSEA) was used to calculate the infiltrating scores of 16 immune cells and 13 immune-related pathways. Then, CIBERSORT algorithm was used to assess the relevance among risk score and 22 immune cells abundance. Subsequently, the differences in expression of immune checkpoints, including PD-1, PD-L1, CTLA4 and LAG3, in ccRCC patients showing different risks were investigated.

### Statistical analysis

All statistical analyses and graphing were performed with the R software (version R-4.1.2) or GraphPad Prism (version 8.0.2). The Student’s t test was adopted to investigate the differences in gene expression between tumor tissues and normal tissues. Spearman correlation analysis was applied to evaluate the relevance between the risk score and the expression of immune checkpoints. *P* value < 0.05 was considered significant. *P* values were showed as: ns, not significant; *, *P*< 0.05; **,*P*< 0.01; ***, *P*< 0.001.

## Results

### Screening of prognostic TGF-β signaling-related genes of ccRCC in the TCGA cohort

**Figure 2:**
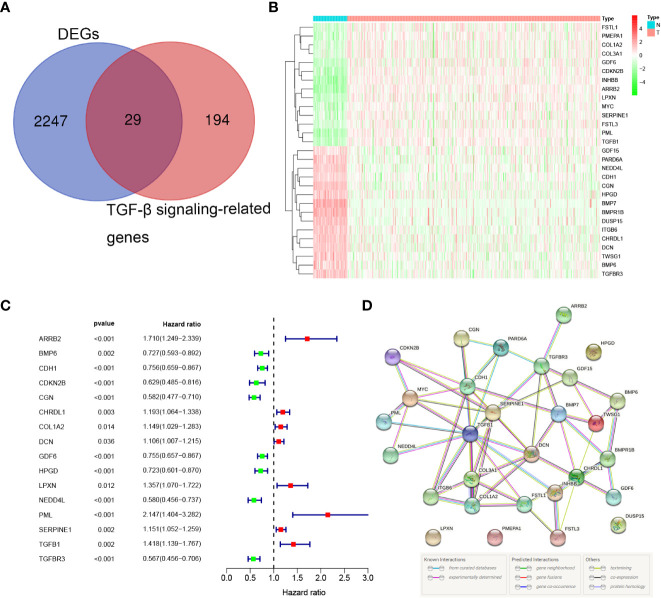
Identification of the prognostic TGF-β signaling-related genes in the TCGA cohort. **(A)** Venn diagram to identify DEGs between normal and tumor tissue. **(B)** The 29 overlapping genes were differently expressed in normal and tumor tissue. **(C)** Forest plots showing the significantly prognostic genes identified with univariate Cox regression analysis based on OS. **(D)** The PPI network downloaded from the STRING database indicated the interactions among candidate genes.

We summarized the flow diagram of this study in **Figure 1**. Among 223 TGF-β signaling-related genes, 29 DEGs were screened in tumor tissues and tumor-adjacent tissues (**Figures 2A, B**). The univariate Cox regression method suggested that 16 of the 29 genes were significantly associated with OS (**Figure 2C**). These 16 TGF-β signaling-related genes were uploaded to STRING to better visualize the interaction network among these genes (**Figure 2D**).

### Development of a prognostic model in the TCGA cohort

**Figure 3:**
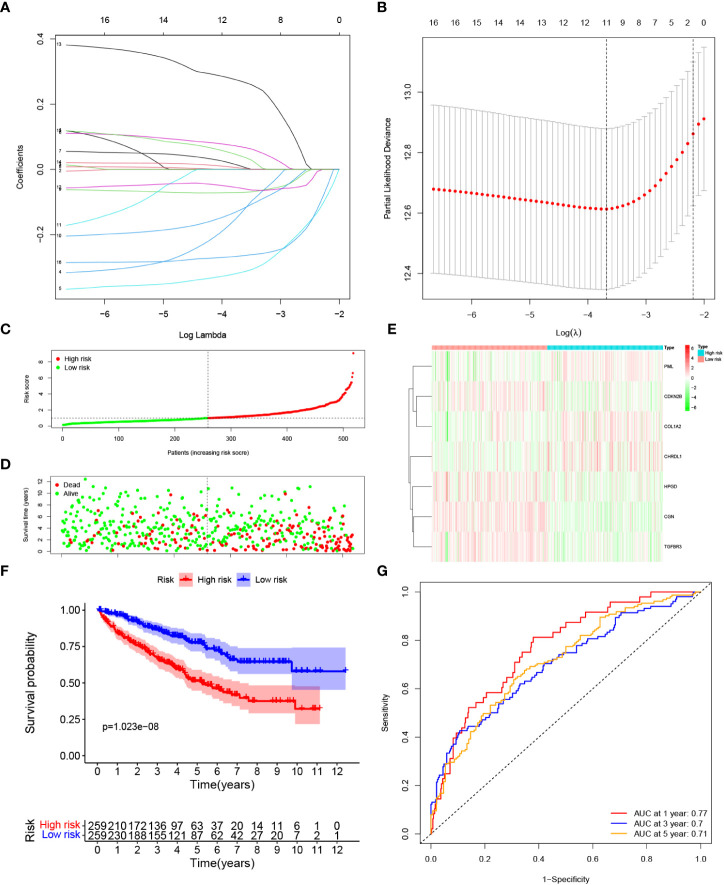
Construction of a prognostic model based on TGF-β signaling-related genes in the TCGA cohort. **(A**, **B)** LASSO Cox regression analysis was applied to screen the key genes. **(C)** The median value and distribution of the risk score. **(D)** The distribution of survival status. **(E)** Expression of seven prognostic genes. **(F)** K-M curves for the OS. **(G)** ROC curve of the prognostic signature.

LASSO Cox regression analysis was conducted to filter out the key genes (**Figures 3A, B**). Then, the multivariate Cox regression method was performed to further screen candidate genes. Finally, 7 genes, PML, CDKN2B, COL1A2, CHRDL1, HPGD, CGN and TGFBR3, were identified as prognostic signature genes. The risk score was measured as follows: risk score = (0.417 × the expression level of PML) + (-0.373 × the expression level of CDKN2B) + (0.109 × the expression level of COL1A2) + (0.104 × the expression level of CHRDL1) + (-0.195 × the expression level of HPGD) + (-0.399 × the expression level of CGN) + (-0.340 × the expression level of TGFBR3). According to the median cut-off value, patients were classified into low risk and high risk groups (**Figure 3C**). Compared with the low risk group, a significantly higher mortality rate were observed in the high risk group (**Figure 3D**). The heatmap result indicated that patients with high risk exhibited high expression levels of PML, COL1A2, and CHRDL1 but low expression of CDKN2B, HPGD, CGN and TGFBR3 (**Figure 3E**). K-M curves suggested that compared with patients with low risk, patients with high risk had a worse OS (*p* < 0.001). (**Figure 3F**). Additionally, the area under the ROC curve (AUC) of the 7-gene signature reached 0.77 at 1 year, 0.7 at 3 years, and 0.71 at 5 years, indicating a favorable predictive efficacy of the prognostic model (**Figure 3G**).

### Validation of the prognostic signature in the E-MTAB-1980 cohort

**Figure 4:**
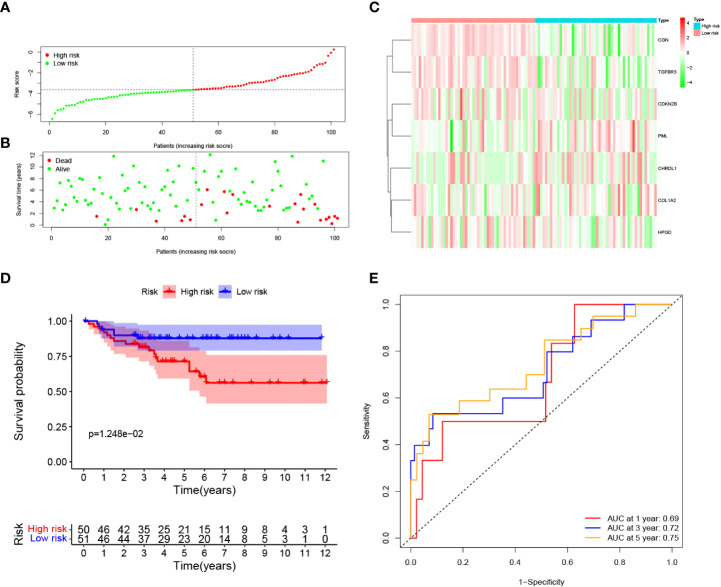
Validation of the prognostic signature in the E-MTAB-1980 dataset. **(A)** Distribution of patients’ risk score, **(B)** Survival status, **(C)** Expression of seven prognostic genes, **(D)** K-M curves for the OS, and **(E)**. ROC curve for evaluating the performance of the prognostic signature in the E-MTAB-1980 dataset.

To evaluate the robustness of the risk model constructed from the TCGA cohort, we categorized patients from E-MTAB-1980 cohort as either high risk group or low risk groups based on the median value calculated by the same risk formula as the TCGA cohort (**Figure 4A**). Patients categorized as high risk group were more likely to die earlier (**Figure 4B**). The expression pattern of the risk model genes were similar to TCGA cohort (**Figure 4C**). The OS of patients in the high risk group was significantly lower than patients in the low risk group (*p* = 0.012). (**Figure 4D**). Additionally, as shown in **Figure 4E**, the AUC of the signature reached 0.69 at 1 year, 0.72 at 3 years, and 0.75 at 5 years, suggesting a better prediction efficacy.

### Independence of the prognostic model and nomogram construction

**Figure 5:**
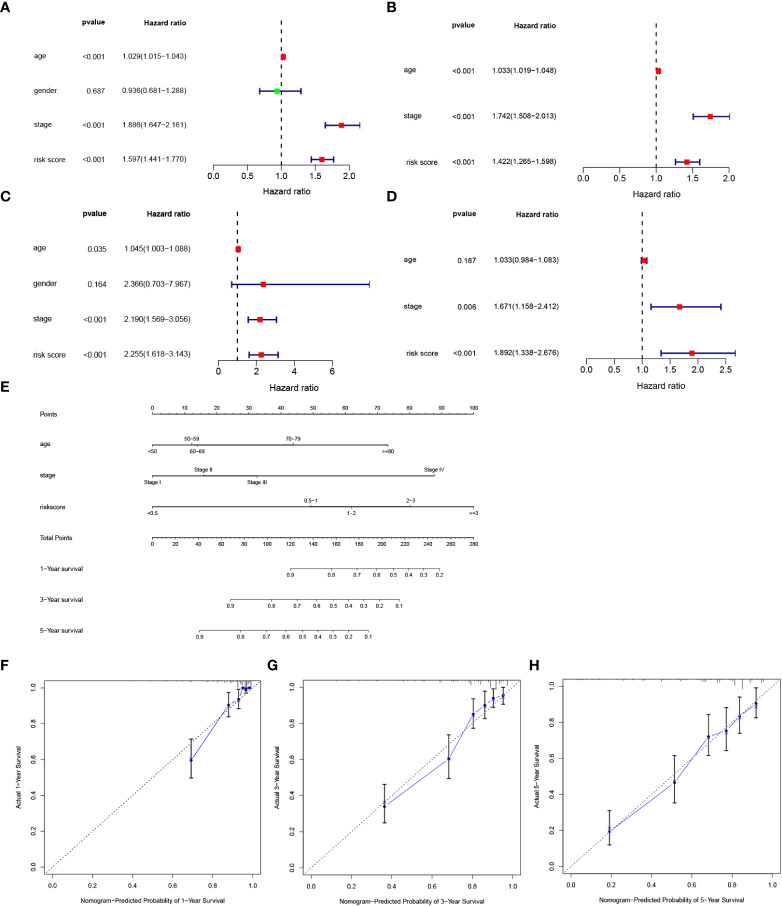
Development of a nomogram predicting OS in ccRCC. **(A**, **B)** Univariate and multivariate cox regression for risk score and clinical features, including age, gender, stage, and risk score in the TCGA cohort. **(C**, **D)** Univariate and multivariate cox regression for risk score and clinical features, including age, gender, stage, and risk score in the E-MTAB-1980 cohort. **(E)** Nomogram integrated age, stage, and riskscore. **(F, H)** Calibration curve for predicting OS at 1, 3 and 5 years.

To clarify whether the signature could serve as an independent prognostic variable for OS, univariate and multivariate Cox regression analyses were performed. Univariate analysis shown that risk score was proven to be strong OS-related factors (TCGA cohort: HR = 1.597, 95% CI =1.441–1.770, *p* < 0.001, **Figure 5A**; E-MTAB-1980 cohort: HR = 2.255, 95% CI= 1.618–3.143, *p* < 0.001, **Figure 5C**). Multivariate analyses revealed that risk score was still a significantly prognostic variable for OS (TCGA cohort: HR = 1.422, 95% CI =1.265–1.598, *p* < 0.001, **Figure 5B**; E-MTAB-1980 cohort: HR = 1.892, 95% CI= 1.338–2.676, *p* < 0.001, **Figure 5D**). Therefore, risk score was confirmed as an independent prognostic factor for OS of ccRCC patients. The independent prognostic factors, namely age, stage and risk score, were utilized to construct a nomogram (**Figure 5E**). The calibration curve revealed that the nomogram presented better predictive performances at 1, 3, and 5 years of survival. **(** **Figures 5F–H** **)**.

### Prognostic model risk score and clinical features

**Figure 6:**
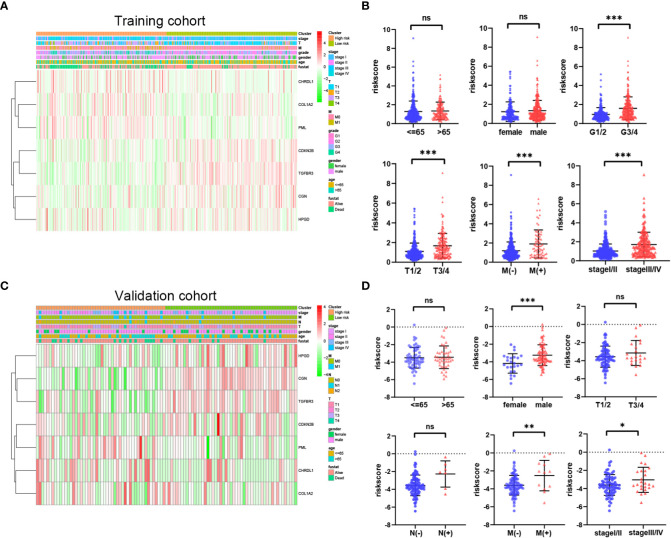
Relationship between riskscore and clinicopathological parameters in the TCGA cohort **(A**, **B)** and E-MTAB-1980 cohort **(C**, **D)**. *P* values were shown as: ns, not significant; *P< 0.05; **P< 0.01; ***P< 0.001.

To investigate the correlation of risk score and clinical features, we analyzed the distribution of risk score values after stratification based on clinicopathological features. As shown in **Figures 6A, B**, the TCGA cohort patients with worse pathological features, including high grade, advanced T stage, metastasis, and advanced TMN stage had an obviously higher risk score. In addition, the E-MTAB-1980 cohort patients with metastasis or advanced TMN stage had an significantly higher risk score (**Figures 6C, D**). In sum, higher risk score were related to higher malignancy in ccRCC.

### Functional enrichment analyses in the TCGA cohort

**Figure 7:**
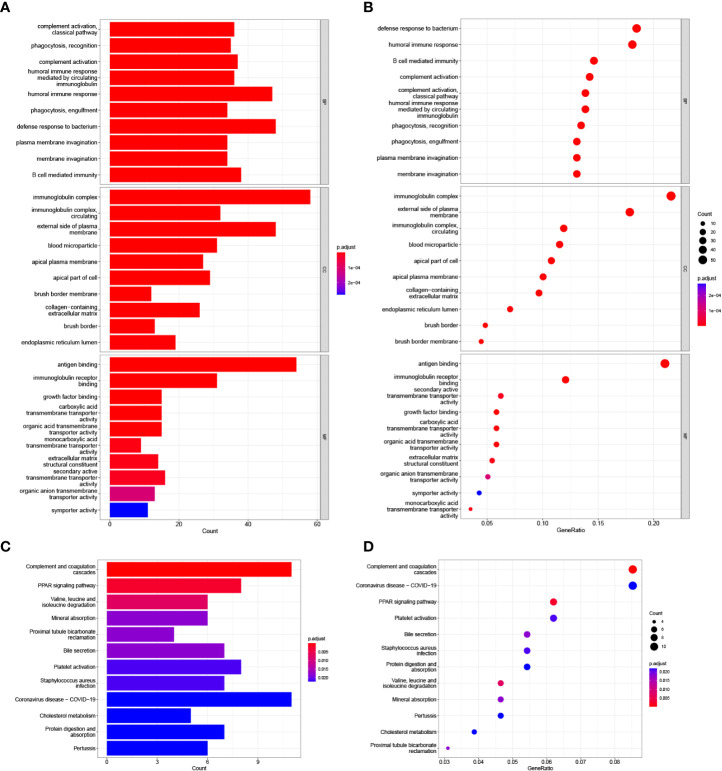
GO and KEGG analysis in the TCGA cohort. **(A**, **B)** GO enrichment analysis. **(C, D)** KEGG enrichment analysis.

GO enrichment and KEGG pathway analyses were utilized to analyze the underlying biological functions and pathways of risk score-related genes. The DEGs between high-risk and low-risk groups was analyzed, and then these DEGs were used for GO enrichment and KEGG pathway analysis. GO analysis revealed that DEGs were enriched in biological processes of the immune responses, including complement activation, humoral immune responses mediated by circulating immunoglobulin, humoral immune responses, B cell mediated immunity (**Figures 7A, B**). KEGG analysis shown that DEGs were correlated with complement and coagulation cascades and PPAR signaling pathway (**Figures 7C, D**).

### Relationship between risk score and immune infiltration landscape in the TCGA cohort

**Figure 8:**
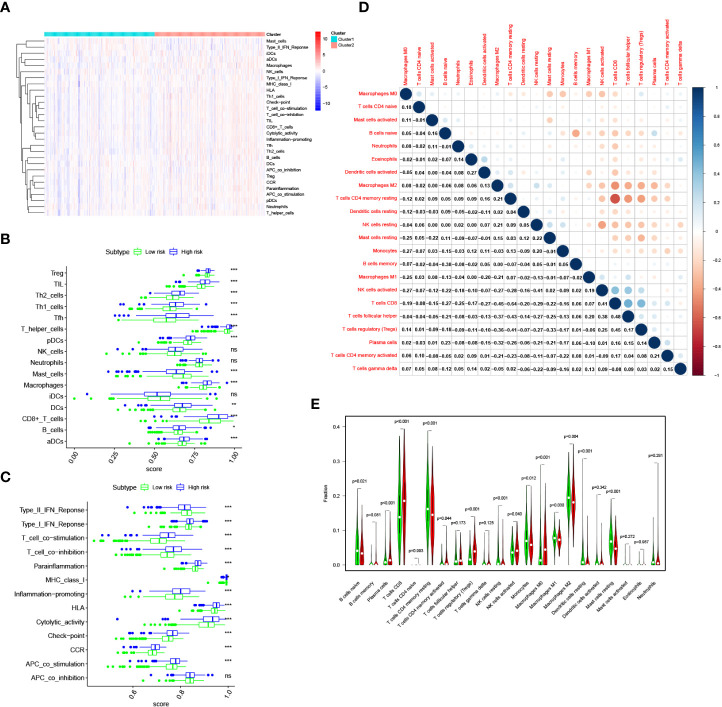
Immune infiltration pattern analysis in the TCGA cohort. **(A)** Relationship heatmap of the riskscore and ssGSEA scores. **(B)** Box plots presenting the scores of immune cells. **(C)** Box plots presenting the scores of immune function. **(D)** CIBERSORT algorithm analysis on correlations between 22 immune cell types. **(E)** CIBERSORT algorithm analysis the distribution of the abundance of immune cell infiltration between the high and low risk score groups. *P* values were shown as: ns, not significant; *P< 0.05; **P< 0.01; ***P< 0.001.

The ssGSEA algorithm was performed to determine the difference of immune activity between the high risk group and low risk group. Immune cell abundance, including CD8+_T_cells, DCs, Macrophages, Mast_cells, pDCs, T_helper_cells, Tfh, Th1_cell, Th2_cells, TIL, B-cells, aDCs, and Treg, were significantly higher in the high risk group (**Figures 8A, B**). Immune function scores, including Type_I_IFN_Reponse, Type_II_IFN_Reponse, T_cell_co-stimulation, T_cell_co-inhibition, Parainflammation, MHC_class_I, Inflammation-promoting, HLA, Cytolytic_activity, check-point, CCR and APC_co_stimulation were stronger in the high risk group than those of in the low risk group (**Figure 8C**). To determine the proportion difference of 22 types of immune cells in the tumor microenvironment between high risk and low risk groups, CIBERSORT algorithm was carried out. Correlations of 22 types of immune cells types are presented in **Figure 8D**. As shown in **Figure 8E**, B cells naive, T cells CD4 memory resting, NK cells resting, Monocytes, macrophages M1, Macrophages M2 and Mast cells resting were significantly higher in the low risk group, while Plasma cells, T cells CD8, T cells CD4 memory activated, T cells regulatory, NK cells activated, and Macrophages M0 were significantly higher in the high risk group.

### Risk model based on TGF-β signaling-related gene could predict the clinical response of immunotherapy

**Figure 9:**
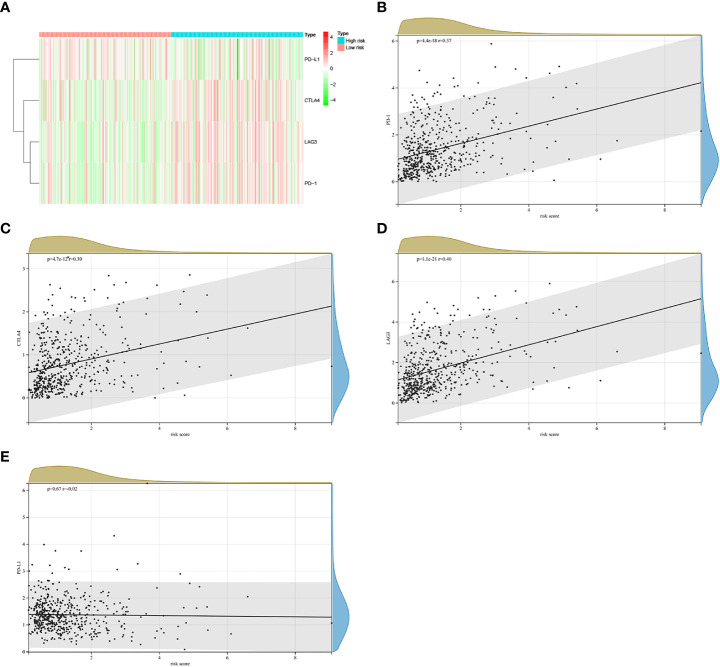
The correlations between riskscore and expression of immune checkpoint molecules in the TCGA cohort. **(A)** Heatmap of immune checkpoint molecules expression, including PD-1, PD-L1, LAG3 and CTLA4. **(B–E)**The relevance between the risk score and the expression of immune checkpoints.

At present, immunotherapy therapy are the main treatment option for advanced ccRCC after targeted therapy failure (6, 17). The difference in the score of immune infiltration landscape in tumor microenvironment between the two risk groups indicated that the difference of immunotherapy effectiveness between the two groups. Common immune molecules, such as PD-1, PD-L1, CTLA4 and LAG3 are essential markers for personalized treatment. In this study, patients in the high risk group had significantly higher expressions of PD-1, CTLA-4, as well as LAG3 and greatly lower expressions of PD-L1 (**Figure 9A**). Further, the Spearman correlation test was used to evaluate the relationship between the risk score and expression of immune checkpoints. We found that the expression of PD-1, CTLA4 and LAG3 were positively correlated with risk score (**Figures 9B–D**), while the expression of PD-L1 were not substantially related to risk score (**Figure 9E**). Combining these results, patients in the high risk group would significantly benefit more after taking immunotherapy than those in the low risk group.

## Discussion

TGF-β signal is a crucial pathway involved in many malignancies initiation and progression. TGF-β signal activation stimulates EMT, facilitating metastasis and chemical resistance (11, 18). In recent years, many studies shown that TGF-β signal gene signature have a favorable capacities for predicting prognosis and responses to treatment of cancer (15, 16, 19). Several studies have reported that TGF-β pathway transduction disorder is very common in ccRCC and that inhibition of TGF-β pathway is considered to be a promising forms of treatment for ccRCC (20, 21). Therefore, a comprehensive exploration of the expression levels of TGF-β signaling-related genes in ccRCC may predict and improve the efficacy of therapy and prognosis of patients.

In this study, we identified and validated a TGF-β signaling-related genes signature in ccRCC and systematically analyzed the signature relationship with risk stratification and prognosis. The OS times of patients with high risk scores was significantly shorter than counterpart with low risk scores. The AUC for OS shown better predictive performance of the gene signature. Independent prognostic analysis confirmed that the risk score had an independent predictive capacity for OS of ccRCC patients. In addition, we found that the high risk scores was significantly associated with unfavorable clinicopathological characteristics, such as higher tumor grade, advanced TMN stage and metastasis.

Our signature consisted of seven TGF-β signaling-related genes, including PML, CDKN2B, COL1A2, CHRDL1, HPGD, CGN and TGFBR3. PML, also known as TRIM19, which was originally found in Acute Promyelocytic Leukemia (22). Cytoplasmic PML can stimulate TGF-β signaling by regulating the signal transduction of the phosphorylation of transcription factors SMAD2/3 (23). Previous study reported that PML act dual roles as oncogenic drivers and tumor suppressors in various malignant tumor (24). A recent study verified that PML was upregulated in triple negative breast cancer and knockdown PML suppressed tumor growth *in vitro* and *in vivo* (25). CDKN2B, also known as P15, belongs to the INK4 family, which has been identified as an inhibitor of cyclin-dependent kinase 4, thus inhibiting cell cycle progression and facilitating cell apoptosis in a variety of human cancers (26, 27). Tu et al. found that CDKN2B inactivation is essential for pancreatic carcinogenesis (28). Previous study revealed that mutation of CDKN2B lead to an increased incidence of renal cell carcinoma (29). COL1A2 (collagen type I alpha 2 chain) is a member of Type I collagen which is the important fibrillary component of extracellular matrix (30). Previous studies suggested that COL1A2 expression was up-regulated in multiple human carcinomas and abnormal increasing expression of COL12A1 was associated with a poor prognosis (31, 32). Dong et al. found that compared with normal tissues, COL1A2 was significantly upregulated in RCC (33). CHRDL1, also known as Chordin-like 1, is an antagonist of bone morphogenetic proteins (BMPs), and BMP signaling involve in several physiological and pathological processes, including cell proliferation, migration and invasion in malignant tumor (34). Wu et al. found that CHRDL1 expression was significantly downregulated in oral squamous cell carcinoma (OSCC). Overexpression of CHRDL1 suppressed OSCC cell metastasis *in vitro* and vivo (35). In breast cancer, CHRDL1 could suppress cell migration and invasion by inhibiting BMP signaling (36). HPGD (15-Hydroxyprostaglandin dehydrogenase), an important enzyme regulating the metabolism of prostaglandins, has been confirmed as a tumor suppressor in many malignancies (37–40). Yao et al. found that HPGD was significant down-regulation in cervical cancer tissues, and overexpression of HPGD suppressed proliferation and migration of cervical cancer cells (41). However, Lehtinen et al. reported that HPGD was significant up-regulation in breast cancers tissues, and high HPGD expression were associated with a poor clinical prognosis of breast cancer (42). CGN (cingulin), a transmembrane protein localized on the cytoplasmic surface of epithelial tight junctions, has been reported as a tumor inhibitor in ovarian cancer and osteosarcoma (43–44). TGFBR3 (transforming growth factor beta receptor 3) is a co‐receptor that bind multiple cytokines of the TGF‐β superfamily (45). TGFBR3 has been confirmed as a tumor suppressor in lung cancer (46), pancreatic cancer (47), prostate cancer (48), and breast cancer (49). Nishida et al. reported that TGFBR3 expression was significantly downregulated in ccRCC, and decreased expression of TGFBR3 was associated with poor clinical prognosis in patients with ccRCC. In addition, silencing TGFBR3 facilitated ccRCC cells growth and metastasis *in vitro* and *in vivo* (50). The above evidence indicated that all the seven TGF-β signaling-related genes correlated with malignant processes of multiple human cancer.

A previous study suggested that TGF-β signaling modulates immune cell infiltration in the tumor microenvironment (51). Immune cell infiltration is closely related to the clinical prognosis of ccRCC (52). According to the ssGSEA algorithm, we found differences in immune infiltration among patients with ccRCC with different risk scores not only in infiltrating scores of immune-cell, but also in infiltrating scores of immunity-related pathways. Patients in high risk group had significantly high infiltrating scores of immune-cell and immunity-related pathways. According to the CIBERSORT algorithm, we found that patients in low risk group had increased infiltration of B cells naive, T cells CD4 memory resting, NK cells resting, Monocytes, macrophages M1, Macrophages M2 and Mast cells resting, while patients in high risk group had increased infiltration Plasma cells, T cells CD8, T cells CD4 memory activated, T cells regulatory, NK cells activated, and Macrophages M0. Previous studies found that T cells regulatory infiltration was associated with poor prognosis in the ccRCC patients (53, 54). High T cells CD8 infiltration level is a poor prognostic factor in the ccRCC patients (55). M1 macrophages play an important role in inflammation induction, antigen presentation and antitumor reactions (56). A study reported that higher Mast cells resting density was associated with favorable outcomes in ccRCC (57). In addition, Zhang et al. reported that compared with high risk group, low risk group had higher abundance of B cells naive, T cells CD4 memory resting, NK cells resting, monocytes and macrophages M2 in the ccRCC patients (58). Therefore, dysregulation of the abundance of immune cell infiltration endowed high risk group an immunosuppressive tumor microenvironment, leading to a poor prognosis.

Cancer immunotherapies significantly improved the clinical prognosis of patients with advanced ccRCC (6, 59). In the TCGA cohort, a significantly distinction in the expression levels of immune checkpoints was found between the two groups. Compared with low risk group, high risk group had significantly higher expression levels of PD-1, CTLA-4, as well as LAG3 and greatly lower expressions of PD-L1. Besides, risk score was positively related to the expression of PD-1, CTLA4 and LAG3. These results indicated that patients in the high risk group would significantly benefit more from immunotherapy. Therefore, this prognostic signature model could be used for predicting the expression level of immune checkpoints and guiding immunotherapy decisions.

Several limitations should be recognized. First, a multi-center prospective study validation should be conducted to increase the evidence level of the prognostic signature model. Second, further experiment are needed to investigate the specific function and mechanisms of the seven genes in future work. Third, constructing a prognostic signature risk model *via* considering a single hallmark datasets might cause the regrettable deletion of several other promising prognostic genes.

## Conclusion

We identified seven prognostic TGF-β signaling-related genes in ccRCC and constructed a robust prognostic signature model that can independently predict the survival outcome. In addition, this prognostic signature was related to the immune cell infiltration and expression of immune checkpoints, which can be used to predict the prognosis and guide immunotherapy decisions.

## Data availability statement

Publicly available datasets were analyzed in this study. This data can be found here: TCGA and the ArrayExpress (E-MTAB-1980) databases.

## Author contributions

LL and XW were responsible for the study design and writing; WX and BG were mainly responsible for data analysis. BF, WC, and LZ were mainly responsible for data collection. LL and XW were responsible for manuscript review and providing constructive comments. All authors contributed to the article and approved the submitted version.

## Supplementary material

Supplementary Table 1 A total of 223 TGF-β signaling-related genes were obtained from the following databases: AmiGO 2 (http://amigo.geneontology.org/amigo/landing), Ensembl Genome Brower (http://grch37.ensembl.org/index.html) and GSEA (http://www.gsea-msigdb.org/gsea/index.jsp).
