# Supplementary material for: Development of a TGF-β signaling-related genes signature to predict clinical prognosis and immunotherapy responses in clear cell renal cell carcinoma
Source: Front Oncol. 2023 Jan 27;13:1124080. doi: 10.3389/fonc.2023.1124080 (PMC9911835; doi:10.3389/fonc.2023.1124080)
Supplement: Supplementary Table 1 — A total of 223 TGF-β signaling-related genes were obtained from the following databases: AmiGO 2 (http://amigo.geneontology.org/amigo/landing), Ensembl Genome Brower (http://grch37.ensembl.org/index.html) and GSEA (http://www.gsea-msigdb.org/gsea/index.jsp). [file Table_1.pdf]

gene
AC023512.1
ACVR1
ACVR1B
ACVR1C
ACVR2A
ACVR2B
ACVRL1
ADAM9
AL353729.1
AMH
AMHR2
APC
APPL1
APPL2
ARHGEF18
ARRB2
ATP1B4
BAMBI
BMP10
BMP2
BMP4
BMP5
BMP6
BMP7
BMP8A
BMP8B
BMPR1A
BMPR1B
BMPR2
CBL
CCNC
CCNK
CCNT1
CCNT2
CDH1
CDH5
CDK8
CDK9
CDKN2B
CER1
CGN
CHRD
CHRDL1
CITED1
CITED2
CLDN5

COL1A2
COL3A1
COMP
CR589904.1
CREB1
CREBBP
CUL1
DCN
DRAP1
DUSP15
DUSP22
E2F4
E2F5
EIF3I
ENG
EP300
F11R
FERMT2
FKBP1A
FNTA
FOS
FOXH1
FSHB
FST
FSTL1
FSTL3
FURIN
FUT8
GCNT2
GDF10
GDF15
GDF2
GDF5
GDF6
GDF7
GDF9
GREM2
HDAC1
HIPK2
HPGD
ID1
ID2
ID3
ID4
IFNG
INHBA
INHBB

INHBC
INHBE
ITGB5
ITGB6
ITGB8
JUN
JUNB
KLF10
LEFTY1
LEFTY2
LPXN
LRRC32
LTBP1
LTBP2
LTBP3
LTBP4
MAP2K1
MAP3K7
MAPK1
MAPK3
MEN1
MIR183
MIR212
MIR26A1
MIR26A2
MIR30A
MSTN
MTMR4
MYC
NCOR1
NCOR2
NEDD4L
NEDD8
NLK
NODAL
NOG
NRROS
NSA2
PARD3
PARD6A
PARP1
PITX2
PMEPA1
PML
PPM1A
PPP1CA
PPP1CB

PPP1CC
PPP1R15A
PPP2CA
PPP2CB
PPP2R1A
PPP2R1B
PRKCZ
PTK2
PTPRK
PXN
RBL1
RBL2
RBX1
RHOA
RNF111
ROCK1
ROCK2
RPS27A
RPS6KB1
RPS6KB2
SERPINE1
SIRT1
SKI
SKIL
SKP1
SMAD1
SMAD2
SMAD3
SMAD4
SMAD5
SMAD6
SMAD7
SMAD9
SMURF1
SMURF2
SNW1
SP1
SRC
STRAP
STUB1
TAB1
TAB2
TAB3
TAB3P1
TBILA
TFDP1
TFDP2

TGFB1
TGFB2
TGFB3
TGFBR1
TGFBR2
TGFBR3
TGFBR3L
TGFBRAP1
TGIF1
TGIF2
THBS1
THBS2
THBS3
THBS4
TNF
TP53
TRIM33
TWSG1
UBA52
UBB
UBC
UBE2D1
UBE2D3
UBE2M
UCHL5
USP15
USP9X
USP9Y
WDR7
WFIKKN2
WWTR1
XPO1
ZFYVE16
ZFYVE9
ZMIZ1
ZYX
